# Sex‐specific genetic regulation of proteomics in cerebrospinal fluid uncovers genetic causes for sex differences in AD and PD

**DOI:** 10.1002/alz70855_107477

**Published:** 2025-12-25

**Authors:** Yun Ju Ju Sung, Anh Do, Soomin Song, Lihua Wang, Daniel Western, Jiseon Kwon, Jigyasha Timsina, Menghan Liu, John P. Budde, Agustin Ruiz, Pau Pastor, Tony Wyss‐Coray, Han Chen, Carlos Cruchaga

**Affiliations:** ^1^ Washington University School of Medicine, Saint Louis, MO, USA; ^2^ NeuroGenomics and Informatics Center, Washington University School of Medicine, St. Louis, MO, USA; ^3^ Washington University School of Medicine, St. Louis, MO, USA; ^4^ Neurogenomics & Informatics Center, St. Louis, MO, USA; ^5^ Ace Alzheimer Center Barcelona – International University of Catalunya (UIC), Barcelona, Spain; ^6^ Memory Disorders Unit, Department of Neurology, Hospital Universitari Mutua de Terrassa, Terrassa, Barcelona, Terrassa, Spain; ^7^ Stanford University, Stanford, CA, USA; ^8^ Human Genetics Center, Department of Epidemiology, Human Genetics and Environmental Sciences, School of Public Health, The University of Texas Health Science Center at Houston, Houston, TX, USA; ^9^ NeuroGenomics & Informatics Center, St. Louis, MO, USA

## Abstract

**Background:**

Clear sex differences exist in AD and PD. Several studies examined genetic regulations for AD phenotypes and gene expression data in a sex‐specific manner, identifying some differences between males and females. In contrasts, although proteins are final effectors of most physiological pathways and important drug targets, sex‐specific regulations for proteins remain vastly understudied. This study therefore aims to investigate sex‐specific genetic regulation of proteomics in neurologically relevant cerebrospinal fluid (CSF).

**Methods:**

We recently identified 3,373 pQTL for 1,886 proteins, many of which were unique to CSF, demonstrating genetic regulation specific to the CSF proteome (Western et al. 2024). Here, we performed a sex‐stratified pQTL (in 1,640 males and 1,713 females, separately) with over 6000 proteins (SomaScan7K) on about 13 million TOPMED imputed autosomal variants. pQTL considered significant at *p* <5×10^‐8^ (in cis) and *p* <3.45×10^‐11^ (in trans) in either sex. We subsequently examined relevance of these pQTL with AD and PD through colocalization and protein‐wise association studies (PWAS).

**Results:**

We identified 1,684 significant pQTLs (1,047 cis and 637 trans) in either sex within 811 genetic loci for 1,488 proteins. There were 384 pQTLs (22.8%) identified only in one sex (215 in males; 169 in females). The male‐specific pQTLs included locus for SNRPD2 on chromosome 19 (β =‐0.76, *p* = 4.63×10^‐8^ in males; β=‐0.04, *p* = 8.38×10^‐1^ in females), whereas the female‐specific pQTLs included locus for IST1 on chr16 (β=0.43, *p* = 4.40×10^‐8^ in females vs β=‐0.02, *p* = 7.89×10^‐1^ in males). The PD locus *LRRK2* on chr12 regulated several proteins (GRN, GPNMB, ITGB2), enriched in microglia, in males only. In addition, the strongest AD risk *APOE* region sex‐specifically regulated multiple proteins relevant to AD (PPP3R1, YWHAZ, PPP3CA, amyloidosis‐related NRXN2, VSNL1, and dementia‐related TIMM8A), enriched in neurons. We identified 22 proteins colocalized and associated with AD risk loci (including ACE in females only and TMEM106B in males only). Similarly, 4 proteins linked to PD loci (including ADAM15 in males).

**Conclusion:**

By performing sex‐stratified pQTL, we uncovered distinctive sex‐specific genetic regulation of CSF proteome. Our findings complement sex‐aware eQTL from GTEx and provide insights on dissecting the underlying biological causes and mechanisms contributing to sex differences in neurodegeneration.

